# Analysis of copy number variation by sequencing in fetuses with nuchal translucency thickening

**DOI:** 10.1002/jcla.23347

**Published:** 2020-04-27

**Authors:** Liubing Lan, Heming Wu, Lingna She, Bosen Zhang, Yanhong He, Dandan Luo, Huaxian Wang, Zhiyuan Zheng

**Affiliations:** ^1^ Prenatal Diagnosis Center Meizhou People's Hospital (Huangtang Hospital) Meizhou Hospital Affiliated to Sun Yat‐sen University Meizhou China; ^2^ Department of Obstetrics Meizhou People's Hospital (Huangtang Hospital) Meizhou Hospital Affiliated to Sun Yat‐sen University Meizhou China; ^3^ Guangdong Provincial Key Laboratory of Precision Medicine and Clinical Translational Research of Hakka Population Meizhou People's Hospital (Huangtang Hospital) Meizhou Academy of Medical Sciences Meizhou Hospital Affiliated to Sun Yat‐sen University Meizhou China; ^4^ Meizhou Municipal Engineering and Technology Research Center for Molecular Diagnostics of Major Genetic Disorders Meizhou People's Hospital (Huangtang Hospital) Meizhou Academy of Medical Sciences Meizhou Hospital Affiliated to Sun Yat‐sen University Meizhou China; ^5^ Center for Precision Medicine Meizhou People's Hospital (Huangtang Hospital) Meizhou Academy of Medical Sciences Meizhou Hospital Affiliated to Sun Yat‐sen University Meizhou China

**Keywords:** chromosomal karyotype, copy number variation sequencing, nuchal translucency

## Abstract

**Objective:**

Copy number variation sequencing (CNV‐seq) technique was used to analyze the genetic etiology of fetuses with increased nuchal translucency (NT).

**Methods:**

A total of 139 women with gestational 11‐14 weeks whose fetuses were detected with increased NT (NT ≥ 2.5 mm) in our hospital from July 2016 to December 2018 were selected. Fetal specimens were performed for karyotyping analysis and CNV sequencing.

**Results:**

According to the nuchal translucency thickness, 2.5‐3.4, 3.5‐4.4, 4.5‐5.4, and more than 5.5 mm, the rates of chromosomal abnormalities were 22.8% (13/57), 30.8% (12/39), 42.1% (8/19), and 62.5% (15/24), respectively. There was significant difference among the incidences of chromosomal abnormalities in four groups (χ^2^ = 37.69, *P* < .01) and the incidences increased with fetal NT thickness. Among 139 cases, there were 36 cases (25.9%) with abnormal chromosome karyotypes. Meanwhile, there were 45 cases (32.3%) with abnormal CNV. In the 12 cases with abnormal CNV and normal chromosome karyotypes, there were 2 cases of pathogenic CNV, 7 cases of CNV with unknown clinical significance, and 3 cases of possibly benign CNV. There was no significant difference in CNV between pregnant women in advanced maternal age and those in normal maternal age (χ^2^ = 1.389, *P* = .239). In the fetus who showed abnormalities in NT and ultrasonography (χ^2^ = 5.13, *P* < .05) and the fetus aborted (χ^2^ = 113.19, *P* < .05), the abnormal rate of CNV was higher with statistically significant difference.

**Conclusion:**

CNV‐seq combined karyotype analysis should be performed simultaneously in fetuses with increased NT, providing a basis for genetic counseling, which is of great significance for prenatal diagnosis.

## 1 INTRODUCTION

Nicolaides et al^1^ first reported in 1992 that NT measurements were used as an ultrasound indicator for chromosome screening in early pregnancy. Increased NT is one of the most sensitive indicators of fetal chromosomal abnormalities in ultrasound screening in early pregnancy. NT refers to the anechoic zone under the skin of the fetal neck, that is, the hydronephrosis between the fetal neck and the skin.^2^ Increased NT is associated with chromosomal abnormalities, genetic syndromes, structural abnormalities, and fetuses or perinatal death and other adverse pregnancy outcomes in early pregnancy. Moreover, the degree of increased NT was directly proportional to the incidence of fetal abnormalities and adverse pregnancy outcomes.^3^, ^4^, ^5^ The prevalence of congenital heart defects (CHD) is in the order of six times higher in fetuses with a NT ≥ 99th percentile than in an unselected population.^6^, ^7^, ^8^ The association of Noonan syndrome with increased NT is clear.^9^, ^10^ What has not been clearly defined is the incidence of other genetic syndromes, congenital defects, and adverse pregnancy and pediatric outcomes in the presence of increased nuchal translucency. Increased NT defined by The Fetal Medicine Foundation as NT ≥ 3.5 mm, as this measurement corresponds to the 99th percentile in the general population (https://fetalmedicine.org).

At present, the most common method for detection chromosomal abnormalities is karyotype analysis. Karyotype analysis is the “gold standard” of cytogenetic detection, but it has a long detection time and low resolution and cannot detect copy number variations (CNVs) no more than 5 Mb. Chromosome microarray analysis (CMA) is a technique for the detection of CNVs in human genome, and it can detect micro‐deletions or micro‐duplications that cannot be detected by traditional karyotype analysis. In recent years, CMA technology has been widely used in prenatal diagnosis.^11^, ^12^, ^13^, ^14^, ^15^, ^16^, ^17^, ^18^, ^19^ However, its high cost and low throughput limit its large‐scale application in prenatal diagnosis. In addition, due to the limited chip probe coverage, some CNVs may not be detected.^20^, ^21^ Due to its high resolution, high throughput, and relatively low cost, the genome copy number variation sequencing (CNV‐seq) technology based on next generation sequencing (NGS) has been gradually developed.^22^, ^23^, ^24^

Meizhou is located in eastern Guangdong Province with a resident population of 5.28 million and an annual birth rate of 12.45‰ (official web site of the Bureau of Health and Family Planning of Meizhou, China). The majority of Meizhou residents are Hakka people. Hakka people are Han Chinese populations that mainly inhabit southern China. The Hakka originated from the southern migration of the central plains in the north. This group has unique hereditary background.

There are no reports about the analysis of copy number variation by sequencing in fetuses with increased NT of Hakka population in Meizhou, China. The purpose of this study is to analyze the chromosomes in fetuses with increased NT by CNV‐seq in order to evaluate its application in prenatal diagnosis.

## 2 MATERIALS AND METHODS

### 2.1 Subjects

From July 2016 to December 2018, fetuses underwent early pregnancy ultrasound screening in Meizhou People's Hospital. Among them, ultrasound screening indicated fetuses with NT ≥ 2.5 mm. Increased NT defined by The Fetal Medicine Foundation as NT ≥ 3.5 mm, as this measurement corresponds to the 99th percentile in the general population (https://fetalmedicine.org). Different studies on the definition of NT thickening mostly use 3.5 mm for cutoff value criticality, but the risk of fetal abnormalities may increase when NT value within 2.5‐2.9 mm, therefore, these fetuses with NT is greater than or equal to 2.5 mm were included in this study.

139 fetuses with prenatal diagnosis (chromosome karyotype analysis and CNV analysis) were finally included in the analysis. All the 139 fetuses were single fetus. The average age of pregnant women was 30 years old. There were 104 patients less than 35 years old and 35 patients more than 35 years old. The study was performed under the guidance of the Declaration of Helsinki and approved by the Ethics Committee of Meizhou People's Hospital (Huangtang Hospital), Meizhou Hospital Affiliated to Sun Yat‐sen University, Guangdong, China.

### 2.2 Measurement of NT by ultrasonic examination

Ultrasonograph was performed with a 2.5‐7 MHz transducer (model GE Voluson E10). According to the method developed by the British fetal several medical foundation^25^: (a) fetal head and buttock length (CRL) 45‐84 mm; (b) supine position was taken for pregnant women, and the median sagittal section of fetal natural flexion was taken for ultrasound images; (c) enlarge the image to 75% of the fetal head and upper chest; (d) distinguish fetal skin from amnion; (e) to measure the maximum thickness of the semi‐transparent tissue between the fetal neck soft tissue and the skin; (f) the scale line should overlap the boundary of thickness of NT; and (g) measure it for 3 times and record its maximum value. NT thickness of 11‐14 weeks fetuses with ≥2.5 mm was defined as Increased NT. It was suggested that pregnant women should conduct fetal chromosome karyotype analysis and further ultrasound examination in the second trimester.

### 2.3 Karyotyping and CNV sequencing

20 mL amniotic fluid was collected by amniocentesis performed under ultrasonography guidance. Half of the amniotic fluid was centrifuged and inoculated in the medium. Each case was inoculated in parallel with two bottles. The cells were harvested and prepared after 10‐11 days of culture in the CO_2_ incubator. Twenty fission images were observed and counted under the microscope after G banding, and 3‐5 karyotypes were analyzed. When abnormalities were found, the count was doubled. The other half of amniotic fluid is centrifuged twice to minimize maternal contamination. DNA was extracted from the cells of amniotic fluid using Qiagen DNA extraction kit (Qiagen) according to the manufacturer's instructions. Detection of the chromosome CNV detection kit (CapitalBio Genomics co., Ltd), according to the instructions: the whole genome DNA of 50 ng was randomly digested into a fragment of about 180 bp, and the end of the broken DNA fragment was supplemented and connected by enzyme reaction. After PCR amplification, the fragment selection and purification were carried out by magnetic bead purification method to remove the interference of primer dimer in the reaction system, so as to obtain the DNA library. CNV Sequencing was performed Life BioelectronSeq 4000 platform (Thermo Fisher).

Copy number gains or losses were compared without in‐house database of copy number variants (CNVs) and with public CNV databases, including GenomicVariants (http://dgv.tcag.ca/dgv/app/home ), Decipher (http://decipher.sanger.ac.uk/), and ClinGenb (https://www.clinicalgenome.org/). If abnormal CNV changes of unknown clinical significance were detected in the amniotic fluid sample, the parental samples were analyzed for the aberration.

### 2.4 Statistical analysis

SPSS statistical software version 21.0 was used for data analysis. The abnormal degree of NT, the age of pregnant women, the presence of other abnormalities, and other CNV abnormalities were analyzed. The data were expressed as the means ± SD. Mann‐Whitney *U* test was used to compare values between groups. Chi‐square and ANOVA tests were used to analyze the differences among the two groups. A value of *P* < .05 was considered as statistically significant.

## 3 RESULTS

### 3.1 The relationship between NT thickness and abnormal CNV detection

Among the 139 samples, 45 were abnormal with CNVs (32.37%). We divided the included patients into four groups with NT value of 1 mm as an interval. 139 fetuses with NT ≥ 2.5 mm at 11‐14 weeks of gestation were divided into four groups, including 2.5‐3.4 mm, 3.5‐4.4 mm, 4.5‐5.4 mm, and ≥5.5 mm groups. With the increase in NT thickness, the ratio of live birth is decreasing, which is statistically significant (χ^2^ = 54.03, *P* < .01). The detection rate of the corresponding abnormal CNV also increases with the increase, statistically significant (χ^2^ = 37.69, *P* < .01), as shown in Table 1.

**TABLE 1:** The relation of CNVs and pregnancy outcome in 139 fetuses with increased nuchal translucency thickness at 11‐14 wk of gestation

NT (mm)	N	Pregnancy outcome				Abnormal CNVs (n %)
		TOP	IUD	Miscarriage	LB (n %)	
2.5‐3.4	57	9	4	2	42 (73.7)	13 (22.8)
3.5‐4.4	39	10	3	1	25 (64.1)	12 (30.8)
4.5‐5.4	19	8	1	0	10 (52.6)	8 (42.1)
≥5.5	24	14	3	1	6 (25.0)	15 (62.5)
Total	139	41	11	4	83 (59.7)	48 (34.5)

Abbreviations: IUD, intrauterine death; LB, live birth; TOP, termination of pregnancy.

### 3.2 CNV‐seq and karyotype analysis

Among the 139 samples, 36 were abnormal with chromosome karyotype. There were 12 cases with abnormal CNVs and normal karyotype, 2 of which were pathogenic CNVs (Table 2). The detected pathogenic CNVs is known as 22q11 micro‐duplication syndrome and 15q11.2 micro‐deletion syndrome. The other ten smaller CNV detected all involved non‐disease genes and none had any disease associations of clinical significance.

**TABLE 2:** A normal CNVs detected in 12 fetuses with normal karyotypes

Case	NT (mm)	Maternal age (y)	Weeks of gestation	Outcome	CNVs result	Genes involved	Phenotype
1	5.6	20	12	LB	Chr1: 247300000‐248280000; 0.98 Mb dup	NLRP3	Unknown
2	3	35	13	LB	ChrX: 5680000‐6760000; 1.08 Mb del	NLGN4X	Unknown
3	3.3	34	14	LB	ChrX: 149140000‐149440000; 0.3 Mb dup	None	Unknown
4	9.8	30	14	TOP	Chr5: 122900000‐123280000; 0.38 Mb dup	CSNK1G3	Unknown
5	3.5	29	12	LB	Chr10: 67780000‐68360000; 0.58 Mb dup	None	Unknown
6	4.6	30	12	TOP	Chr17: 140000‐840000; 0.7 Mb dup	VPS53, GEMIN4, RPH3AL, C17orf97, FAM101B, FAM57A, GLOD4, RNMTL1, NXN	Unknown
8	3.6	27	12	IUD	Chr22: 18960000‐21460000; 2.5 Mb dup	COMT, PRODH, SLC25A1, RTN4R, SCARF2, SNAP29, TBX1, LZTR1, GP1BB, SERPIND1	22q11 microduplication syndrome
9	3.4	23	13	TOP	Chr15: 22760000‐23100000; 0.34 Mb del	TUBGCP2, NIPA1, NIPA2, CYFIP1	15q11.2 delection syndrome
10	3.2	37	13	LB	Chr15: 22760000‐23080000; 0.32 Mb dup	NIPA1	Unknown
11	2.6	24	12	LB	Chr6: 104180000‐104880000; 0.70 Mb dup	None	Unknown
12	3.3	32	13	LB	ChrX: 6480000‐8120000; 1.64 Mb dup	STS	Unknown

CNV‐seq correctly detected 33 of the 36 gross chromosomal abnormalities identified by karyotyping, including trisomy 21 (n = 14), trisomy 18 (n = 6), trisomy 13 (n = 3), 47, XXX (n = 2), 45, X (n = 6), and 47, XXY (n = 1), and detailed data were listed in Table 3. The two samples that had discordant CNV‐seq and karyotyping results involved 92, XXXX and 46, Xn, inv(9) (p13q13).

**TABLE 3:** CNVs detected in 36 fetuses with an anormal karyotype

Case (num.)	CNVs result	Karyotype
I (2)	47, XXX	47, XXX
II (6)	45, X0	45, X0
III (14)	47, Xn, +21	47, Xn, +21
IV (6)	47, Xn, +18	47, Xn, +18
V (1)	47, Xn, +16	47, Xn, +16
VI (1)	47, XXY	47, XXY
VII (3)	47, Xn, +13	47, Xn, +13
VIII (1)	Normal	92, XXXX
IX (1)	Normal	46, Xn, inv(9) (p13q13)

### 3.3 Statistical analysis of CNV results under different factors

There was no statistically significant difference between elderly and non‐elderly pregnant women with abnormal CNV (χ^2^ = 1.389, *P* = .239). The difference between NT abnormality alone and NT with other ultrasound abnormalities was statistically significance (χ^2^ = 5.128, *P* = .024). There was statistically significant difference in different pregnancy outcomes (χ^2^ = 113.187, *P* < .01) (Table 4).

**TABLE 4:** Analysis of CNVs results under different factors

Factors	CNVs results		χ^2^	*P*
	Normal (n)	Abnormal (n, %)		
advanced maternal age (≥35 y)	21	14 (40.0%)	1.389	.239
Non‐advanced maternal age (<35 y)	71	33 (31.7%)		
Single NT abnormity	43	14 (24.6%)	5.128	.024
Other findings on ultrasound	49	33 (40.2%)		
Induced labor	12	42 (77.8%)	113.187	<.001
Live birth	82	3 (3.5%)		

## 4 DISCUSSION

As early as 2004, the British fetal foundation recommended that prenatal screening in early pregnancy include age, serological screening, and NT, which could lead to a screening rate of 80%‐90% for Down syndrome. In recent years, the role of NT examination in chromosome aneuploidy screening has also been affirmed. Meanwhile, NT values above 99th percentile for gestational age seem to be associated with increased rates of chromosomal/structural abnormalities and adverse perinatal outcomes.^26^

In this study, 139 cases of NT thickened fetus were studied and 45 fetal abnormal CNVs were detected. According to the thickness of NT, subjects were divided into 4 groups, and it was found that with the increase of thickness, the proportion of abnormal CNV also increased, and the induction rate also increased. There was no statistical difference in abnormal CNV ratio between elderly pregnant women and non‐elderly pregnant women, suggesting that NT was an age‐independent prenatal screening indicator. Those with increased NT combined with other ultrasonic abnormal indicators had higher CNV abnormal rate and higher induction rate than those with increased NT alone. Provide guidance for prenatal diagnosis and counseling of NT abnormalities.

In addition to 33 cases of chromosomal abnormalities greater than 5M (including aneuploidy), CNV‐seq found an additional 12 cases of micro‐deletions and micro‐duplications. One patient was 22q11.2 micro‐duplication syndrome, and 1 case was 15q11 micro‐deletion syndrome. 22q11.2 chromosome micro‐duplication syndrome refers to a common 3 Mb or 1.5 Mb with proximal tandem repeat. About 70% of patients had inherited the disease from parents who were not affected or had mild symptoms. The syndrome is usually mild and highly heterogeneous. And patients can range from asymptomatic to mental/learning disabilities, psychomotor retardation, growth retardation and/or hypotonia. 15q11.2 deletion syndrome is caused by a 300‐500 kb deletion of the BP1 to BP2 region in 15q11.2, which contains four non‐imprinted genes, *TUBGCP2*, *NIPA1*, *NIPA2*, and *CYFIP1*. The syndrome has a variety of phenotypes and is incompletely dominant. The absence of this region increases susceptibility to neuropsychiatric or neurodevelopmental problems, including delayed psychomotor development, delayed speech development, autism spectrum disorder, attention deficit hyperactivity disorder, obsessive‐compulsive disorder, and epilepsy. In the studies of other scholars, there have also been reports of single or multiple abnormal ultrasound and the detection of pathogenic CNVs that cannot be detected by traditional karyotype.^27^, ^28^, ^29^, ^30^ Our study found 7 cases of clinically unknown CNVs and 3 cases of possibly benign CNVs. Subsequently, CNV‐seq analysis was conducted on the parents of the 7 fetuses, and the results indicated that the 5 fetuses were inherited from their parents with normal performance, which was determined as benign CNVs, and the other 2 were newly developed, with unclear clinical significance. This is the difficulty and limitation of chromosome CNVs analysis. The above cases, especially for neonates with clinically unknown CNVs, need long‐term follow‐up to determine whether they are pathogenic.

With the rapid development of genomics, the OMIM database is adding 1.5 genes per day on average. Chromosome microarray analysis (CMA) probes cannot be designed to keep up with this rate of update, which inevitably results in missed detection of known pathogenic regions. And the high cost and low throughput of CMA limit its large‐scale application in prenatal diagnosis. Since CNV‐seq was first reported in 2009 that it could be used for accurate analysis of chromosomal copy number variation,^31^ more and more studies have confirmed its reliability in the detection of CNVs. CNV‐seq can be used as an alternative method of CMA and even has advantages in the detection of small fragments of genome abnormalities.^24^, ^32^, ^33^ CNV‐seq based on NGS technology conducted sequencing analysis on samples, compared the sequencing results with the human reference genome, and found CNVs through biological information analysis. A high‐throughput sequencer can simultaneously perform non‐invasive prenatal screening (NIPT) and CNV‐seq detection, effectively utilizing the cost of the equipment in the laboratory space. The cost of CNV‐seq for one case is only a few hundred yuan (RMB). And it is time‐efficient and prospective in the detection of copy number variation. In addition, the initial amount of DNA required by CNV‐seq in clinical application is as low as 10 ng, which is 5% of the amount of DNA required by CMA, and the detection sensitivity is higher. Therefore, in prenatal diagnosis, CNV‐seq technology has the advantages of rapid, accurate, high resolution, high throughput, and relatively low cost.

There are two cases with normal CNVs but abnormal karyotypes, 92, XXXX and 46, Xn, inv(9) (p13q13) in our study. Due to the principle of technical analysis, chromosome euploidy abnormalities, balanced translocation and inversion of chromosomes, and single diploidy cannot be detected by CNV‐seq technology. Therefore, we suggest that CNV‐seq combined karyotype analysis should be performed simultaneously in patients with chromosomal diseases and high‐risk groups. In this way, more detailed and accurate clinical diagnosis can be provided for patients, thus providing a basis for genetic counseling and fertility guidance.

## 5 CONCLUSION

In conclusion, NT is an effective means of prenatal diagnosis and screening. For the prenatal diagnosis of NT thickened fetus, the chromosomal karyotype analysis plus CNV‐seq detection mode will be able to detect chromosomal micro‐deletions and micro‐duplications at the whole genome level, which is of great significance for prenatal diagnosis.

## AUTHORS' CONTRIBUTIONS

Liubing Lan and Zhiyuan Zheng conceived and designed the experiments. Lingna She, Bosen Zhang, Yanhong He, Dandan Luo recruited subjects and collected clinical data. Zhiyuan Zheng and Huaxian Wang conducted the laboratory testing. Heming Wu helped to analyze the data. Zhiyuan Zheng prepared the manuscript. Heming Wu and Zhiyuan Zheng reviewed the manuscript.

## ETHICAL APPROVAL

This study was conducted on the basis of the Declaration of Helsinki and was supported by the Ethics Committee of the Meizhou People's Hospital.
